# Comparative plaque- and liquid-based phage susceptibility testing of *Pseudomonas aeruginosa* isolates in a cystic fibrosis randomized clinical trial

**DOI:** 10.1128/jcm.00875-26

**Published:** 2026-10-27

**Authors:** Christina A. Koscianski, Wanying Shao, Lauren Komarow, Krupa Parmar, Kerryl E. Greenwood-Quaintance, Maria Souli, Mikeljon P. Nikolich, Andrey A. Filippov, Robert T. Schooley, Scott R. Evans, David Pride, Daria Van Tyne, Henry F. Chambers, Vance G. Fowler, Pranita D. Tamma, Robin Patel, on behalf of the Antibacterial Resistance Leadership Group

**Affiliations:** 1 Division of Clinical Microbiology, Department of Laboratory Medicine and Pathology, Mayo Clinic 6915, Rochester, Minnesota, USA; 2 The Biostatistics Center, Milken Institute School of Public Health, The George Washington University 50430, Washington, DC, USA; 3 Duke Clinical Research Institute, Duke University Medical Center 169142, Durham, North Carolina, USA; 4 Wound Infections Department, Bacterial Diseases Branch, Center for Infectious Disease Research, Walter Reed Army Institute of Research 8394, Silver Spring, Maryland, USA; 5 Division of Infectious Diseases and Global Public Health, Department of Medicine, UC San Diego 8784, La Jolla, California, USA; 6 Department of Biostatistics and Bioinformatics, Milken Institute School of Public Health, The George Washington University 50430, Washington, DC, USA; 7 Division of Laboratory and Genomic Medicine, Department of Pathology, UC San Diego 8784, La Jolla, California, USA; 8 Division of Infectious Diseases, University of Pittsburgh 271847, Pittsburgh, Pennsylvania, USA; 9 Division of HIV, Infectious Diseases & Global Medicine at Zuckerberg San Francisco General, Department of Medicine, University of California, San Francisco, California, USA; 10 Division of Infectious Diseases, Department of Pediatrics, University of Pennsylvania Perelman School of Medicine 215865, Philadelphia, Pennsylvania, USA; 11 Division of Public Health, Infectious Diseases, and Occupational Medicine, Department of Medicine, Mayo Clinic 6915, Rochester, Minnesota, USA; Children's Hospital Los Angeles, Los Angeles, California, USA

**Keywords:** cystic fibrosis, phage susceptibility testing, *Pseudomonas aeruginosa*

## Abstract

The optimal phage susceptibility testing (PST) method is unclear. Here, two PST methods were compared using *Pseudomonas aeruginosa* isolates recovered from patients with cystic fibrosis enrolled in a placebo-controlled clinical trial of a single intravenous dose of a four-phage cocktail (WRAIR-PAM-CF1). In total, 508 *P. aeruginosa* sputum isolates were evaluated, including isolates collected before or after phage or placebo treatment, isolates from the same trial participants over time, and isolates of different colony morphologies from the same participants at the same time point. Susceptibility to phages PaWRA01Φ11, PaWRA01Φ39, PaWRA02Φ83, and PaWRA02Φ87 and a cocktail of all four phages was assessed using a plaque assay (PA) and a Biolog Omnilog-based liquid assay (LA) with the same bacterial inoculum. A total of 170/508 isolates (33%) and 195/508 isolates (38%) were identified as susceptible to the cocktail by PA and LA, respectively. Among the susceptible isolates, 169/170 (99%; PA) and 187/195 (96%; LA) also tested susceptible to at least one of the four phages in the cocktail. Susceptible percent agreement between the PA and LA ranged from 76% to 100% and non-susceptible percent agreement ranged from 89% to 100% for all phages studied and the cocktail. Phage PaWRA02Φ83 demonstrated the greatest individual activity, with 164/508 (32%) and 188/508 (37%) isolates testing susceptible by PA and LA, respectively. The four-phage cocktail provided numerically broader coverage of *P. aeruginosa* (i.e., had the highest susceptibility rate) than did any single phage. There was high concordance between PST results by PA and LA.

**IMPORTANCE:**

Standardization of phage susceptibility testing (PST) methods is essential to support phage therapy trials and any future clinical implementation. There are no standardized PST methods or interpretive criteria for PST. In this study, PST was performed by a plaque assay and a Biolog Omnilog-based liquid assay on *Pseudomonas aeruginosa* isolates recovered from cystic fibrosis patients enrolled in a clinical trial. Susceptibility of 508 isolates to four phages and a cocktail of all four was evaluated. There was high concordance between susceptibility assessed by plaque and liquid assays and numerically broader coverage of *P. aeruginosa* by the cocktail compared to any single phage. This manuscript should be of interest to those performing PST.

## INTRODUCTION

The antibiotic resistance crisis is driving a strong interest in bacteriophage (phage) therapy. To support both phage therapy clinical trials and, ultimately, use in clinical practice, standardized methods to measure phage susceptibility are needed. Whereas methods for conventional antibiotic susceptibility testing are standardized, yielding similar results across laboratories, there are no standardized phage susceptibility testing (PST) methods and no standardized interpretive criteria for the results of PST (1). Moreover, when phage cocktails are administered, approaches to evaluating potential synergy or antagonism between phages may be helpful and operationally, evaluation of a cocktail may be easier than testing its components.

Although there are multiple possible methods to perform PST, two common approaches are plaque assay (PA) and liquid assay (LA) methods. PA methods assess plaque formation as an indicator of phage activity. LA methods assess bacterial growth or metabolism in the presence of phage in a liquid media; delayed rises in bacterial optical density measured at 600 nm (OD_600_) or bacterial respiration measured using redox dyes, because of phage infection, may be assessed at relatively high throughput, such as using 96-well plates.

The Antibacterial Resistance Leadership Group (ARLG) recently completed a randomized clinical trial of a four-phage cocktail (WRAIR-PAM-CF1) in *Pseudomonas aeruginosa*-colonized cystic fibrosis patients (NCT05453578) (2). Prior to performing the trial, PST was performed using the four phages (PaWRA01Φ11, PaWRA01Φ39, PaWRA02Φ83, and PaWRA02Φ87) and the cocktail at three centers, two using a LA and the third performing a PA, with 145 *P. aeruginosa* isolates, including 46 from patients with cystic fibrosis (3). Results indicated that the reproducibility of PST methods needed further development (3), and therefore, both PA and LA were performed in the above-mentioned clinical trial, with resolution of discrepant results. Here, results of the concurrently performed PA and LA in support of the clinical trial are reported, including cocktail PST results.

## MATERIALS AND METHODS

### Collection and isolation of test isolates

Susceptibility to four individual phages (PaWRA01Φ11 [4], PaWRA01Φ39 [5], PaWRA02Φ83 [6], and PaWRA02Φ87 [6]) and a cocktail (WRAIR-PAM-CF1 [6]) containing all four phages was assessed against 511 *P. aeruginosa* isolates from sputum of cystic fibrosis participants enrolled in the ARLG PHAGE trial, a phase 1b/2, multicenter, randomized, double-blind, placebo-controlled trial of a single intravenous dose of a four-phage cocktail (NCT05453578) (2). Sputum was collected prior to and 2 h after phage or placebo administration at the baseline visit (day 1) and at follow-up visits on days 2, 5 ± 2, 8 + 3, 12 + 7, and 30 ± 7. After collection, sputum samples were shipped on ice from 17 study sites to the University of California San Diego for *P. aeruginosa* isolation. Within 48 h after collection, 10 mg of sputum was added to 1 mL of Sputolysin (MilliporeSigma), vortexed for 15 s, and incubated at room temperature for 15 min. Samples were then serially diluted with Dulbecco’s phosphate-buffered saline (PBS) (10^−1^–10^−4^) and plated on trypticase soy agar with 5% sheep blood and MacConkey agar. Plates were incubated at 37°C in 5% CO_2_ overnight or until growth was adequate for colony enumeration. For plates with multiple colony morphologies, individual morphologies were separately analyzed. Isolates were confirmed as being *P. aeruginosa* by matrix-assisted laser desorption ionization-time-of-flight mass spectrometry using the MALDI Biotyper Microflex LT/SH platform (Bruker Scientific). Confirmed *P. aeruginosa* isolates were inoculated into microbanks (Pro-Lab Diagnostics) and stored at −80°C before being shipped on dry ice to the Mayo Clinic (Rochester, Minnesota) for PST and biobanking in the ARLG biorepository.

### Study phages and host strains

Phages and phage host strains (described in Parmar et al. [3]) were provided by Adaptive Phage Therapeutics (Rockville, MD) and stored at −80°C until use. Phages PaWRA01Φ11 and PaWRA01Φ39 used the host strain PaWRA01, while phages PaWRA02Φ83 and PaWRA02Φ87 used the host strain PaWRA02.

### Phage preparation (titer and normalization) for phage susceptibility testing

Bacterial hosts were inoculated onto sheep blood agar and incubated at 37°C overnight, following which each host was inoculated into trypticase soy broth (TSB) and incubated at 37°C on an orbital shaker at 175 rpm until log phase was reached. Phages were removed from the freezer and thawed on wet ice. Phages were serially diluted 10-fold (10^−1^–10^−8^) in PBS without Ca^2+^ or Mg^2+^ (Cytiva) with dilutions of 10^−5^–10^−8^ of each phage used for spotting. Log-phase bacterial hosts (100 µL) were added to 5 mL of molten 0.75% trypticase soy agar (TSA), mixed, poured on solidified 1.5% TSA plates, and allowed to cool. Then, 10 µL of diluted phage was spotted in triplicate onto host double-layer agar plates and allowed to dry at room temperature. Plates were incubated overnight at 37°C, and plaques were counted the following day. Plaques were averaged between replicates, and plaque-forming units/mL (pfu/mL) were calculated. Phages were diluted in PBS to a final working stock of 1 × 10^8^ pfu/mL for PST. Phage stocks were retained at 4°C for up to 1 month, after which a new stock was prepared. The cocktail was freshly prepared before each round of testing at a ratio of 1:1:1:1 of each individual phage.

### Phage susceptibility testing overview

Phage susceptibility was assessed by two methods, a PA and LA (using a Biolog Omnilog instrument to assess bacterial metabolic activity). The two assays were performed together on the same testing day using the same phage stock and the same bacterial inoculum. Bacterial isolates were streaked on sheep blood agar and incubated for 24–48 h at 37°C. Once growth was adequate, each isolate was added to TSB and incubated at 37°C on an orbital shaker until log-phase was reached. Cultures were then adjusted to an optical density (OD) measured at wavelength 600 nm (OD_600_) of 0.085–0.115 and used for both PA and LA.

### Plaque assay method

An OD-adjusted bacterial culture (100 µL) was added to 5 mL of tempered 0.75% TSA, mixed, poured onto a solidified 1.5% TSA plate, and allowed to cool. Each phage and the phage cocktail were serially diluted (10^−1^–10^−8^) in PBS. Following dilution, 2 µL of each phage or cocktail dilution was spotted onto cooled double-layer agar plates. Plates were allowed to dry and incubated at 37°C overnight or until a full bacterial lawn was observed. Plates were removed from the incubator, and zones of clearance and plaque quality were assessed using a 0–4 scale (7): 0, no lysis or phage activity observed; 1, lysis from without, very faint spots seen in low dilutions with no countable plaques; 2, clear or turbid spots with tiny plaques, which were countable or uncountable; 3, clear spots with small- to medium-sized plaques occurring in the highest dilutions; and 4, large, clear plaques occurring in the highest phage dilutions (Fig. S1). A score of 0–1 was classified as non-susceptible, and 2–4 as susceptible. Host strains PaWRA01 and PaWRA02 were included in each round of testing as quality control strains; there were no quality control failures.

### Liquid assay method

The LA was performed as described in reference 3. Briefly, freshly prepared 1% (vol/vol) Biolog tetrazolium dye D (Biolog, Inc.) diluted in TSB was filter sterilized with a Steriflip 0.22 µm vacuum filter (MilliporeSigma). Biolog 96-well microtiter plates were inoculated with 80 µL of TSB/dye media, 10 µL of phage or phage cocktail (final concentration, 1 × 10^6^ pfu/mL), and 10 µL of OD-adjusted bacteria (final concentration, 1 × 10^5^ colony-forming units/mL). The following quality control tests were included in each run of testing: phages and their respective host (positive control), test media (tetrazolium/TSB alone negative control), bacteria (bacteria alone growth control), and phages (phage sterility control). Plates were placed into an Omnilog at 37°C, and measurements were taken every 15 min over 48 h. Data were converted to comma-separated value (CSV) files using Biolog Data Analysis Software (version 1.7, Biolog Inc.) and analyzed using PhageSelect (Adaptive Phage Therapeutics Inc.), a web-based software, to produce hold times, in hours, determined by taking the time of bacterial growth inhibition by phage compared to the bacterium without phage. A hold time of <4 h was classified as non-susceptible, and ≥4 h as susceptible, recognizing that there is not a standardized breakpoint for defining phage susceptibility. When a control failed (six instances), the entire round of testing (i.e., PA and LA) was repeated.

### Test concordance

Isolates with concordant results between PA and LA at initial testing underwent no further testing. Isolates with discordant results between PA and LA at initial testing were subjected to an additional two rounds of testing by PA and LA on two separate days. For isolates with discordance between initial PA and LA, the final assay result for each assay was determined by concordance of two out of three tests. Three isolates had insufficient growth, in both the PA and LA, to determine phage susceptibility and were therefore excluded from analysis.

### Statistical analysis

For the initial and final test results, susceptible percent agreement (SPA) and non-susceptible percent agreement (NSPA) were estimated along with 95% score confidence intervals (CIs) (8). Analyses were conducted considering the PA as the reference test and repeated considering the LA as the reference test. SPA was estimated by the proportion of isolates classified as susceptible by the test under evaluation among those classified as susceptible by the test used as the reference. NSPA was estimated by the proportion of isolates that were classified as non-susceptible by the test under evaluation among those classified as non-susceptible by the test used as the reference. Coverage was defined as the percentage of susceptible isolates. Analyses were performed using SAS software, version 9.4 (SAS Institute Inc., Cary, North Carolina). Figures were created using R, version 4.2.1.

## RESULTS

### Susceptibility to individual phages and the four-phage cocktail

**Fig 1:**
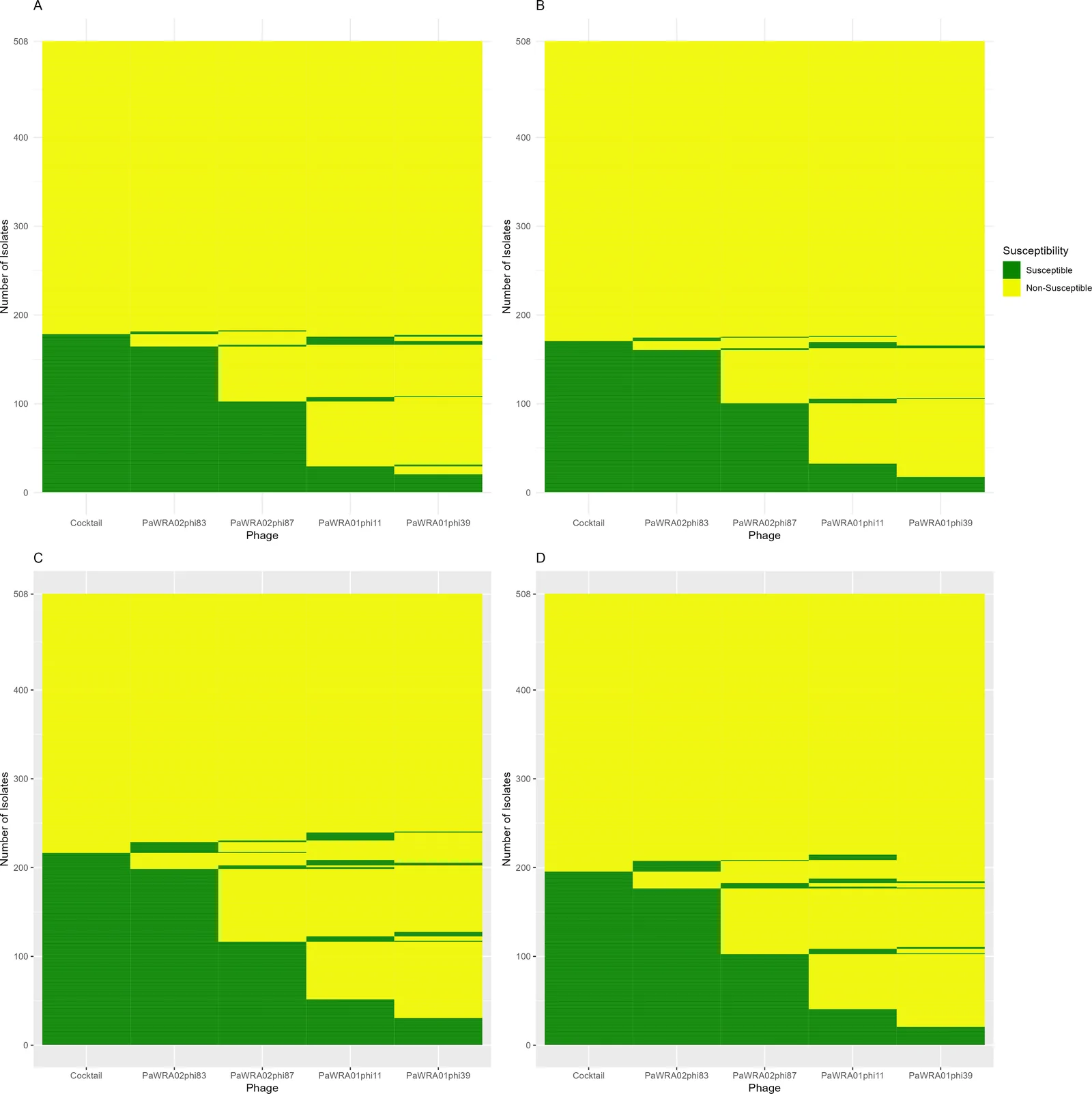
Pattern plots displaying susceptibility patterns of 508 individual *Pseudomonas aeruginosa* isolates to PaWRA01Φ11, PaWRA01Φ39, PaWRA02Φ83, PaWRA02Φ87, and the four-phage cocktail. Color coding is based on the susceptibility of an isolate to a phage or phage cocktail: green—phage susceptible; yellow—phage non-susceptible. (**A**) Initial plaque assay, (**B**) final plaque assay, (**C**) initial liquid assay, and (**D**) final liquid assay. Interpretation is explained here in detail for panel **A**. For panel **A**, among the 178 isolates that were susceptible (green) to the four-phage cocktail, 20 (11%) were susceptible to all four individual phages; 11 (6%) were susceptible to three individual phages (9 were susceptible to PaWRA01Φ11, PaWRA02Φ83, and PaWRA02Φ87; 2 were susceptible to PaWRA01Φ39, PaWRA02Φ83, and PaWRA02Φ87); 81 (46%) were susceptible to two individual phages (4 were susceptible to both PaWRA01Φ11 and PaWRA01Φ39; 5 were susceptible to both PaWRA01Φ11 and PaWRA02Φ83; 1 was susceptible to both PaWRA01Φ39 and PaWRA02Φ83; 71 were susceptible to both PaWRA02Φ83 and PaWRA02Φ87); 65 (37%) were susceptible to one individual phage (5 were susceptible to PaWRA01Φ11; 2 were susceptible to PaWRA01Φ39; 56 were susceptible to PaWRA02Φ83; 2 were susceptible to PaWRA02Φ87); and 1 (1%) was susceptible to none of the four individual phages. Among the 330 isolates that were non-susceptible (yellow) to the four-phage cocktail, no isolates (0%) were susceptible to two, three, or all four individual phages, 4 (1%) were susceptible to one individual phage (3 were susceptible to PaWRA02Φ83, 1 was susceptible to PaWRA02Φ87), and 326 (99%) were susceptible to none of the four individual phages.

Results of PST for PA and LA across individual phages and the cocktail are summarized in Table 1, with susceptibility patterns shown in Fig. 1. The cocktail demonstrated the highest overall activity, with 33% and 38% of isolates classified as susceptible by final PA and LA, respectively. Among individual phages, isolates showed the greatest susceptibility to phage PaWRA02Φ83 (32% PA, 37% LA), followed by PaWRA02Φ87 (20% PA, 21% LA) and PaWRA01Φ11 (9% PA, 12% LA). Isolates showed the lowest susceptibility to phage PaWRA01Φ39, with 4% and 5% classified as susceptible by PA and LA, respectively.

**TABLE 1:** Frequency of phage susceptibility by plaque and liquid assay methods for 508 *Pseudomonas aeruginosa* isolates using phages PaWRA01Φ11, PaWRA01Φ39, PaWRA02Φ83, and PaWRA02Φ87 and the cocktail of the four phages^*a*^

Assay	Susceptibility result	PaWRA01Φ11 (*N* = 508)	PaWRA01Φ39 (*N* = 508)	PaWRA02Φ83 (*N* = 508)	PaWRA02Φ87 (*N* = 508)	Phage cocktail (*N* = 508)
Initial plaque assay	S	43 (8%)	29 (6%)	167 (33%)	105 (21%)	178 (35%)
	NS	465 (92%)	479 (94%)	341 (67%)	403 (79%)	330 (65%)
Initial liquid assay	S	74 (15%)	40 (8%)	210 (41%)	123 (24%)	216 (43%)
	NS	434 (85%)	468 (92%)	298 (59%)	385 (76%)	292 (57%)
Final plaque assay	S	45 (9%)	21 (4%)	164 (32%)	103 (20%)	170 (33%)
	NS	463 (91%)	487 (96%)	344 (68%)	405 (80%)	338 (67%)
Final liquid assay	S	59 (12%)	26 (5%)	188 (37%)	109 (21%)	195 (38%)
	NS	449 (88%)	482 (95%)	320 (63%)	399 (79%)	313 (62%)

^*a*^ S, susceptible; NS, non-susceptible.

### Comparison of individual phage to phage cocktail susceptibility

When isolates were classified as susceptible to the cocktail, 99% and 96% of isolates by final PA and final LA, respectively, also showed susceptibility to one to four of the individual phages (Table 2). One isolate was susceptible to the cocktail but not to any individual phage by final PA and eight isolates by final LA testing. When isolates were classified as non-susceptible to the cocktail, 98% and 94% of isolates by final PA and final LA, respectively, also showed non-susceptibility for all phages tested individually (Table 2). Six isolates were susceptible to an individual phage but not to the cocktail by final PA, and 19 isolates by final LA testing.

**TABLE 2:** Number of susceptible individual phages when the cocktail tested susceptible or non-susceptible in initial and final plaque and liquid assays^*a*^

Assay	Susceptibility result	Number of susceptible or non-susceptible phage(s)				
		0	1	2	3	4
Initial plaque assay	S (*N* = 178)	1 (1%)	65 (37%)	81 (46%)	11 (6%)	20 (11%)
	NS (*N* = 330)	326 (99%)	4 (1%)	0 (0%)	0 (0%)	0 (0%)
Initial liquid assay	S (*N* = 216)	8 (4%)	76 (35%)	80 (37%)	22 (10%)	30 (14%)
	NS (*N* = 292)	268 (92%)	23 (8%)	1 (0%)	0 (0%)	0 (0%)
Final plaque assay	S (*N* = 170)	1 (1%)	60 (35%)	77 (45%)	15 (9%)	17 (10%)
	NS (*N* = 338)	332 (98%)	6 (2%)	0 (0%)	0 (0%)	0 (0%)
Final liquid assay	S (*N* = 195)	8 (4%)	73 (37%)	72 (37%)	22 (11%)	20 (10%)
	NS (*N* = 313)	294 (94%)	19 (6%)	0 (0%)	0 (0%)	0 (0%)

^*a*^ S, susceptible; NS, non-susceptible.

### Initial versus final assay agreement

The initial assay agreement compared the initial PA to the initial LA, without resolution of discordants between the test results. Final assay agreement compared the PA and LA after two additional repeat tests of discordant isolates, with the final PA and LA result determined by concordance of two out of three tests. Overall initial assay agreement was 94%, 93%, 89%, 86%, and 81% for PaWRA01Φ39, PaWRA01Φ11, PaWRA02Φ87, PaWRA02Φ83, and the cocktail, respectively; final assay agreement increased to 99%, 97%, 94%, 93%, and 90%, respectively (Table 3).

**TABLE 3:** Overall assay agreement between plaque and liquid assays for 508 *Pseudomonas aeruginosa* isolates against phages PaWRA01Φ11, PaWRA01Φ39, PaWRA02Φ83, and PaWRA02Φ87 and the cocktail of the four phages^*a*^

Assay agreement		PaWRA01Φ11 (*N* = 508)	PaWRA01Φ39 (*N* = 508)	PaWRA02Φ83 (*N* = 508)	PaWRA02Φ87 (*N* = 508)	Phage cocktail (*N* = 508)
Initial/Final	Yes/No					
Initial assay agreement Plaque assay-liquid assay	Yes	471 (93%)	475 (94%)	435 (86%)	454 (89%)	412 (81%)
	S-S	40 (8%)	18 (4%)	152 (30%)	87 (17%)	149 (29%)
	NS-NS	431 (85%)	457 (90%)	283 (56%)	367 (72%)	263 (52%)
	No	37 (7%)	33 (6%)	73 (14%)	54 (11%)	96 (19%)
	S-NS	3 (1%)	11 (2%)	15 (3%)	18 (4%)	29 (6%)
	NS-S	34 (7%)	22 (4%)	58 (11%)	36 (7%)	67 (13%)
Final assay agreement Plaque assay-liquid assay	Yes	494 (97%)	501 (99%)	470 (93%)	478 (94%)	459 (90%)
	S-S	45 (9%)	20 (4%)	157 (31%)	91 (18%)	158 (31%)
	NS-NS	449 (88%)	481 (95%)	313 (62%)	387 (76%)	301 (59%)
	No	14 (3%)	7 (1%)	38 (7%)	30 (6%)	49 (10%)
	S-NS	0 (0%)	1 (0%)	7 (1%)	12 (2%)	12 (2%)
	NS-S	14 (3%)	6 (1%)	31 (6%)	18 (4%)	37 (7%)

^*a*^ S, susceptible; NS, non-susceptible.

### Final susceptible and non-susceptible percent agreement

**Fig 2:**
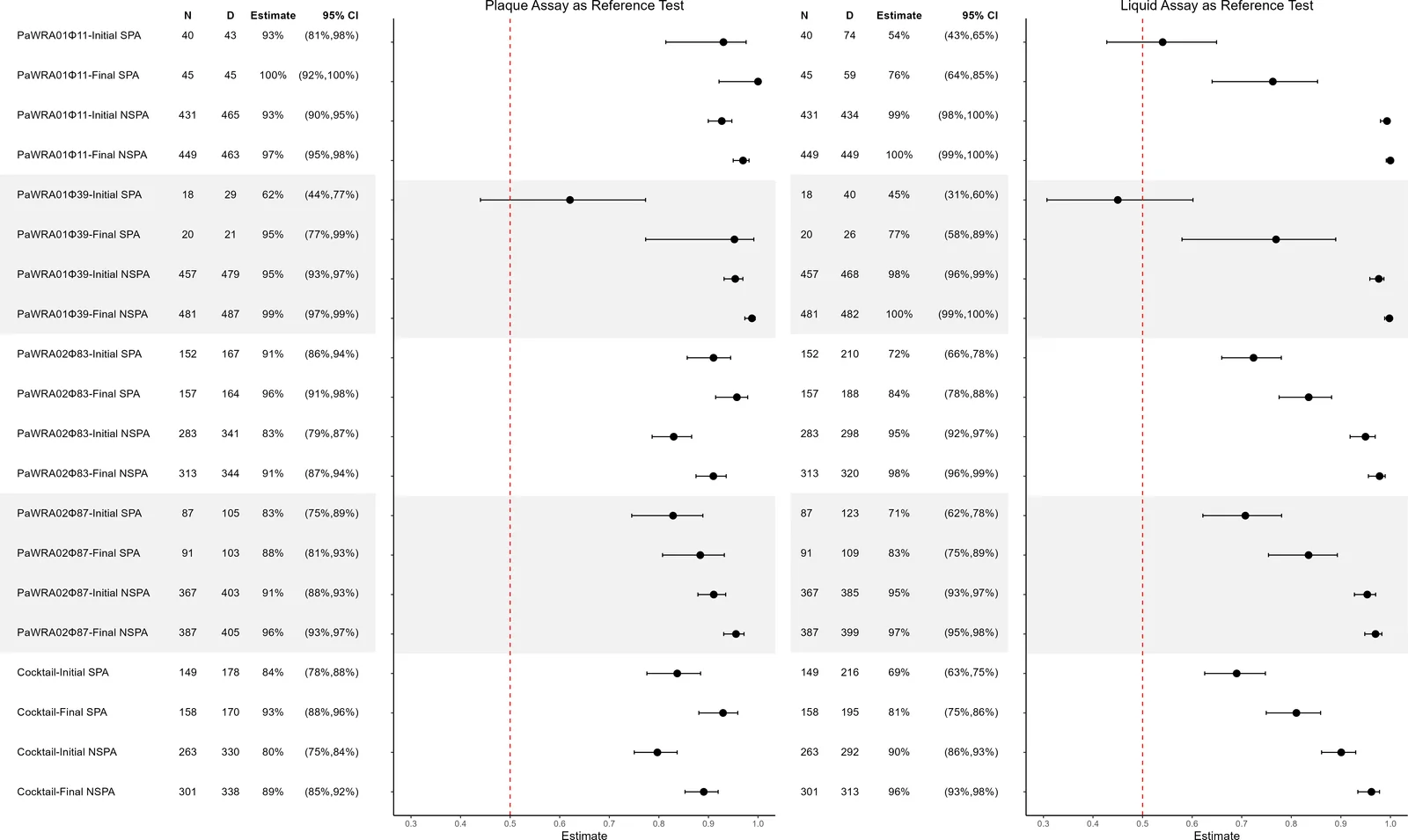
Forest plots showing susceptible percent agreement (SPA) and non-susceptible percent agreement (NSPA) of 508 *Pseudomonas aeruginosa* isolates to phages PaWRA01Φ11, PaWRA01Φ39, PaWRA02Φ83, and PaWRA02Φ87 and the four-phage cocktail with the plaque or liquid assay used as the reference test. N, numerator of SPA or NSPA estimates; D, denominator of SPA or NSPA estimates.

When the final PA was used as the reference test, the final LA SPA ranged from 88% to 100%, and the NSPA ranged from 89% to 99% across all four phages and the cocktail (Table 4, Fig. 2). Phage PaWRA01Φ11 had 100% (95% CI, 92–100%) SPA; PaWRA02Φ87 had the lowest SPA (88% [95% CI, 81–93%]). Phage PaWRA01Φ39 had the highest NSPA (99% [95% CI, 97–99%]); the cocktail had the lowest NSPA (89% [95% CI, 85–92%]).

**TABLE 4:** Susceptible and non-susceptible percent agreement of initial and final liquid assay results with the plaque assay used as the reference test for 508 *Pseudomonas aeruginosa* isolates against phages PaWRA01Φ11, PaWRA01Φ39, PaWRA02Φ83, and PaWRA02Φ87 and the four-phage cocktail^*a*^

Phage	Liquid assay	Test outcome	Reference test (plaque assay) – S	Reference test (plaque assay) – NS	Susceptible percent agreement (95% CI)	Non-susceptible percent agreement (95% CI)
PaWRA01Φ11	Initial	S	40	34	93% (81–98%)	93% (90–95%)
		NS	3	431		
	Final	S	45	14	100% (92–100%)	97% (95–98%)
		NS	0	449		
PaWRA01Φ39	Initial	S	18	22	62% (44–77%)	95% (93–97%)
		NS	11	457		
	Final	S	20	6	95% (77–99%)	99% (97–99%)
		NS	1	481		
PaWRA02Φ83	Initial	S	152	58	91% (86–94%)	83% (79–87%)
		NS	15	283		
	Final	S	157	31	96% (91–98%)	91% (87–94%)
		NS	7	313		
PaWRA02Φ87	Initial	S	87	36	83% (75–89%)	91% (88–93%)
		NS	18	367		
	Final	S	91	18	88% (81–93%)	96% (93–97%)
		NS	12	387		
Cocktail	Initial	S	149	67	84% (78–88%)	80% (75–84%)
		NS	29	263		
	Final	S	158	37	93% (88–96%)	89% (85–92%)
		NS	12	301		

^*a*^ The initial liquid assay was compared to the results of the initial plaque assay as the reference test. The final liquid assay was compared to the results of the final plaque assay as the reference test. S, susceptible; NS, non-susceptible.

When the final LA was used as the reference test, the final PA SPA ranged from 76% to 84% and NSPA ranged from 96% to 100% across all four phages and the cocktail (Table 5, Fig. 2). Phage PaWRA02Φ83 had the highest SPA overall at 84% (95% CI, 78–88%), and phage PaWRA01Φ11 had the lowest SPA at 76% (95% CI, 64–85%). Phage PaWRA01Φ11 had 100% (95% CI, 99–100%) NSPA. The cocktail had the lowest overall NSPA, at 96% (95% CI, 93–98%).

**TABLE 5:** Susceptible and non-susceptible percent agreement of initial and final plaque assay results with the liquid assay used as the reference test for 508 *Pseudomonas aeruginosa* isolates against phages PaWRA01Φ11, PaWRA01Φ39, PaWRA02Φ83, and PaWRA02Φ87 and the four-phage cocktail^*a*^

Phage	Plaque assay	Test outcome	Reference test (liquid assay) – S	Reference test (liquid assay) – NS	Susceptible percent agreement (95% CI)	Non-susceptible percent agreement (95% CI)
PaWRA01Φ11	Initial	S	40	3	54% (43-65%)	99% (98–100%)
		NS	34	431		
	Final	S	45	0	76% (64–85%)	100% (99–100%)
		NS	14	449		
PaWRA01Φ39	Initial	S	18	11	45% (31–60%)	98% (96–99%)
		NS	22	457		
	Final	S	20	1	77% (58–89%)	100% (99–100%)
		NS	6	481		
PaWRA02Φ83	Initial	S	152	58	72% (66–78%)	95% (92–97%)
		NS	15	283		
	Final	S	157	7	84% (78–88%)	98% (96–99%)
		NS	31	313		
PaWRA02Φ87	Initial	S	87	18	71% (62–78%)	95% (93–97%)
		NS	36	367		
	Final	S	91	12	83% (75–89%)	97% (95–98%)
		NS	18	387		
Cocktail	Initial	S	149	29	69% (63–75%)	90% (86–93%)
		NS	67	263		
	Final	S	158	12	81% (75–86%)	96% (93–98%)
		NS	37	301		

^*a*^ The initial plaque assay was compared to the results of the initial liquid assay as the reference test. The final plaque assay was compared to the results of the final liquid assay as the reference test. S, susceptible; NS, non-susceptible.

SPA was higher overall when the PA was used as the reference compared to when the LA was used as the reference. NSPA was slightly higher when the LA was used as the reference.

### Initial compared to final susceptible and non-susceptible percent agreement

When the PA was used as the reference test, assay SPA increased when replicate testing was performed for discordant results, with initial and final values ranging from 62% to 93% and 88% to 100%, respectively (Table 4, Fig. 2). Likewise, NSPA increased when replicate testing was performed for discordant results, with initial and final values ranging from 80% to 95% and 89% to 99%, respectively.

When the LA was used as the reference test, SPA increased when replicate testing was performed for discordant results, with initial and final values ranging from 45% to 72% and 76% to 84%, respectively (Table 5, Fig. 2). Likewise, NSPA increased when replicate testing was performed for discordant results, with initial and final values ranging from 90% to 99% and 96% to 100%, respectively.

### Analysis of multiple *P. aeruginosa* colony morphologies in sputum culture

A total of 106 sputa had two colony morphologies of *P. aeruginosa,* and 16 had three morphologies. Among the 106 sputa that had a second colony morphology, 46 isolate pairs (43%) had the same PST result. In total, 35/46 (76%) of the second morphology isolates with the same susceptibility as the first morphology were non-susceptible for all phages and the cocktail by final PA and final LA. When the first and second morphologies were susceptible, all isolates (11/46) were susceptible to phage PaWRA02Φ83 and the cocktail by PA and LA, with 6/11 isolates also susceptible to phage PaWRA02Φ87 (Table S1). Of the 16 sputa with three colony morphologies, 5 had a third colony morphology isolate with the same PST result as either the first or second colony morphology isolate, one of which was non-susceptible by final PA or final LA. When the third morphology was phage susceptible and had the same PST result as the first or second morphology, 4/5 were susceptible to phage PaWRA02Φ83 and the cocktail by final PA and final LA, with three isolates also susceptible to phage PaWRA02Φ87 and one also susceptible to phage PaWRA01Φ11 (Table S1). Two of the third morphologies had the same phage susceptibility as both the first and second morphologies. Two sputa containing three colony morphologies had two phage-susceptible morphologies and one phage-non-susceptible morphology (Table S1). One sputa containing three colony morphologies had one phage-susceptible morphology and two phage-non-susceptible morphologies (Table S1).

## DISCUSSION

This study reports isolate susceptibility to four individual phages and a cocktail containing all four phages against 508 *P. aeruginosa* isolates by two PST methods. The four-phage cocktail provided the broadest numerical phage coverage overall, compared to any individual phage. There is no current standard PST method. The PA is a commonly used technique for PST but is labor intensive and prone to subjectivity, potentially making it challenging to implement in clinical laboratories (9, 10). A standardized “high throughput” assay would be ideal for routine PST; a LA is a consideration in this regard (11–13).

A notable finding was the limited coverage of study isolates by the four individual phages and the cocktail, which may be attributable to many factors, including possibly inclusion of isolates collected both before and after phage therapy (i.e., bacterial resistance to the trial phages may have developed). A prior study evaluating the same phages against 145 *P. aeruginosa* isolates (including 46 from patients with cystic fibrosis) reported broader coverage (3). The initial LA performed on the 145 isolates at the same testing site that performed testing in the current study showed isolate susceptibility of 12%, 31%, 29%, 32%, and 59% for PaWRA01Φ39, PaWRA01Φ11, PaWRA02Φ87, PaWRA02Φ83, and the cocktail, respectively; the PA performed on the 46 cystic fibrosis isolates showed 13%, 30%, 39%, 46%, and 59% susceptibility for PaWRA01Φ39, PaWRA01Φ11, PaWRA02Φ87, PaWRA02Φ83, and the cocktail, respectively (3). In contrast, for the initial LA results in the current study, the respective values were 8%, 15%, 24%, 41%, and 43% for PaWRA01Φ39, PaWRA01Φ11, PaWRA02Φ87, PaWRA02Φ83, and the cocktail, and the initial PA results in the current study were 6%, 8%, 21%, 33%, and 35% for PaWRA01Φ39, PaWRA01Φ11, PaWRA02Φ87, PaWRA02Φ83, and the cocktail. The cystic fibrosis isolates in the prior study (3) are a distinct population compared to those studied here. To advance phage therapy for patients with cystic fibrosis, broader or alternative phage combinations may be needed.

There are several limitations to this study. First, there are currently no clinically validated phage susceptibility breakpoints (1), and the term “susceptible” can itself be questioned (1); an alternative could be to use “active” and “inactive” (1), but this does not remove the limitation. The findings of “susceptibility” reported here are *in vitro* observations; whether they translate to human phage therapy is unknown (1). *In vitro* “susceptibility” may change if different breakpoints were to be applied. Only a hold time of 4 h was evaluated for susceptibility in the LA based on results of a prior study (3); a reduced or prolonged hold time cutoff might yield different results. Also, there is no reference standard for phage susceptibility testing. Second, this work was performed as a substudy of an as-yet-unpublished clinical trial (2); association of PST with phage therapy response, specific colony morphologies, results of bacterial whole-genome sequencing, and/or results of antimicrobial susceptibility testing are not addressed here. Third, clonality of isolates has not been determined; isolates of different morphologies from the same subject or even isolates between subjects could be related. Fourth, only *P. aeruginosa* isolated from cystic fibrosis sputum was studied. Susceptibility to the individual phages and phage cocktail may vary by disease state from which isolates are derived and possibly over time and by geography. Fifth, the cocktail was tested at a final concentration of one-fourth that of each individual phage (although the total phage amount was the same); susceptibility to the cocktail might vary if equal amounts of phages were to be tested. Sixth, the Biolog Omnilog instrument used for the LA has recently been retired from production, and Adaptive Phage Therapeutics was acquired by BiomX Inc.

In conclusion, the four-phage cocktail, WRAIR-PAM-CF1, provided numerically broader coverage of *P. aeruginosa* isolates from cystic fibrosis trial participants than did any individual phage, whether tested by PA or LA, and generally recapitulated the combined results of individual PST. Notably, fewer than half of the study isolates tested were susceptible to the cocktail. There was high concordance between susceptibility as assessed by PA and LA.

## SUPPLEMENTAL MATERIAL

Fig. S1 jcm.00875-26-s0001.tiff Plaque quality assessment scale.

Supplemental table and legend jcm.00875-26-s0002.docx Table S1 and legend for Fig. S1.

ASM does not own the copyrights to Supplemental Material that may be linked to, or accessed through, an article. The authors have granted ASM a non-exclusive, world-wide license to publish the Supplemental Material files. Please contact the corresponding author directly for reuse.
