# Supplementary material for: Comparative plaque- and liquid-based phage susceptibility testing of Pseudomonas aeruginosa isolates in a cystic fibrosis randomized clinical trial
Source: J Clin Microbiol. 2026 Oct 27;64(11):e00875-26. doi: 10.1128/jcm.00875-26 (PMC13753802; doi:10.1128/jcm.00875-26)
Supplement: Supplementary file 1 [file jcm.00875-26-s0002.docx]

**Supplementary Table 1. Final phage susceptibility of multiple morphology sputa with a matching susceptibility profile to another morphology against phages PaWRA01Φ11, PaWRA01Φ39, PaWRA02Φ83, PaWRA02Φ87, and the four-phage cocktail.**

| **First morphology** | **Susceptible phages** | | **Second morphology** | **Susceptible phages** | | **Third morphology** | **Susceptible phages** | |
| --- | --- | --- | --- | --- | --- | --- | --- | --- |
|  | **PA** | **LA** |  | **PA** | **LA** |  | **PA** | **LA** |
| 1A | 83, 87, C | 83, 87, C | 1B | 83, 87, C | 83, 87, C |  | | |
| 2A | 83, 87, C | 83, 87, C | 2B | 83, 87, C | 83, 87, C |  |  |  |
| 3A | 83, 87, C | 83, 87, C | 3B | 83, 87, C | 83, 87, C |  |  |  |
| 4A | 83, 87, C | 83, 87, C | 4B | 83, 87, C | 83, 87, C | 4C | 83, 87, C | 83, 87, C |
| 5A | 83, 87, C | 83, 87, C | 5B | 83, 87, C | 83, 87, C | 5C | 83, 87, C | 83, 87, C |
| 6A | 83, 87, C | 83, 87, C | 6B | 83, 87, C | 83, 87, C |  | | |
| 7A | 83, C | 83, C | 7B | 83, C | 83, C |  |  |  |
| 8A | 83, C | 83, C | 8B | 83, C | 83, C |  |  |  |
| 9A | 83, C | 83, C | 9B | 83, C | 83, C |  |  |  |
| 10A | 83, C | 83, C | 10B | 83, C | 83, C |  |  |  |
| 11A | 83, C | 83, C | 11B | 83, C | 83, C |  |  |  |
| 12A | NS | NS | 12B | 83, C | 83, C | 12C | 83, C | 83, C |
| 13A | NS | NS | 13B | 11, 83, 87, C | 11, 83, 87, C | 13C | 11, 83, 87, C | 11, 83,87, C |
| 14A | NS | NS | 14B | 83, 87, C | 83, 87, C | 14C | NS | NS |

*NS, non-susceptible; 11, PaWRA01Φ11; 83, PaWRA02Φ83; 87, PaWRA02Φ87; C, cocktail

**Supplementary Figure 1. Plaque quality assessment scale. (A) Score of 0, indicating no observed lysis. (B) Score of 1, indicating lysis from without. (C) Score of 2, indicating clear or turbid spots with tiny countable or uncountable plaques. (D) Score of 3, indicating clear spots with small- to medium-sized plaques at the highest dilutions. (E) Score of 4, indicating large clear spots and plaques at the highest dilutions.**
